# College and Hospital Notes

**Published:** 1901-01

**Authors:** 


### COLLEGE AND HOSPITAL NOTES.

PLANS have been filed and ground broken for the new four-story Emergency Hospital at Eagle and Pine Streets. When completed it will be one of the most perfectly equipped and imposing emergency hospitals in this country, and one which will be highly creditable to the City of Buffalo.

The building is to be 142 by 60 feet. The material is to be steel, with porous terra cotta arches, and brick with Medina stone trimmings for the exterior. An ambulance entrance runs along the end of the building. The basement is planned principally for reception work essential to such a hospital. The main features are the reception and operating rooms. This floor is on a level with the ambulance entrance, so that patients may be discharged from the wagons with ease. Three operating rooms for minor surgery or preliminary examinations are conveniently placed. There is, besides, the pharmacy, dining room and kitchen.

On the first floor there are two wards besides the reception rooms, and a couple of additional operating rooms. The diet kitchen is situated here also. On the next floor there are more wards and a chapel. The third floor is cut up into wards and private rooms for patients. A feature of the third floor is the operating amphitheatre, which is arranged according to the latest requirements of modern surgery. It occupies the best position as to light and is separated from the wards and private rooms, and within easy reach of the elevator. There is a diet kitchen here also.

The steel frame is entirely enclosed by porous terra cotta. The floors are cement over terra cotta arches with marble mosaic. The wainscoting is marble. This style of construction extends through the operating rooms, wards, reception rooms, halls, kitchens, etc. Only the doors, door frames and window frames are of wood.

The roof of the building is constructed on the roof-garden plan. The elevator opens on the roof and patients and nurses can use this space, 142x55, for exercise during the summer. The building is to cost $60,000.

---

The new German Hospital, on Jefferson Street, was formally opened Sunday evening, December 2, 1900, when a splendid bazaar was inaugurated. The building is a substantial one in every respect, and is ample for the needs which it is intended to meet. Mr. William Lautz made the opening address in German. The three floors of the building were filled with booths, in which were attendants dressed in nurses' costume. The bazaar continued two weeks and was a splendid success.

The hospital association was incorporated in 1895 and for several years has conducted a dispensary at No. 621 Genesee Street, where over 2,000 persons have been treated yearly. The dispensary will be continued. The officers of the association are William Simon, president; Dr. Charles H. W. Auel, vice-president; Jacob J. Lang, treasurer; Charles H. North, secretary; George F. Lehmann, assistant secretary; trustees, Charles A. Lautz, Jacob Stern, Otto Reinecke, E. G. S. Miller and Charles Duchmann.

The hospital will be ready to receive patients about January 1st.

---

The alumni of the University of Pennsylvania who reside in Western New York have formed an alumni association. Mr. Charles C. Harrison, Provost of the University, accompanied by Dr. H. C. Wood and a number of the trustees came to Buffalo to assist the local committee, consisting of Drs. Ernest Wende, W. H. Heath, Chauncey Pelton Smith and Truman J. Martin to complete the organisation.

The first annual dinner was given at the Saturn Club, December 17, 1900.

Hon. T. Guilford Smith, Regent of the University of the State of New York, gave a reception at the University Club in honor of Provost Harrison during the latter's stay in Buffalo. There are about 250 alumni in Western New York, 30 of whom live in Buffalo.

---

THE Almshouse committee of the board of supervisors recently examined the architect's revised plans for the consumption hospital to be built next to the Almshouse, to take the place of the one destroyed by fire last year. The amended plans call for a two-story building to cost, it is estimated, about $45,000. On the lower floor there will be accommodations for the 40 male consumptives and there will be ample quarters on the upper floor for the 16 female consumptives now at the County Hospital.

---

PLANS for a cancer hospital in connection with the Buffalo General Hospital were filed with the bureau of buildings last month. The plans were drawn by Mr. George Cary, the architect.

They provide for a three-story fire-proof building suitable for a complete modern hospital. It will be used as an annex of the Buffalo General Hospital and will be erected near that institution.

